# Quantifying error in occurrence data: Comparing the data quality of iNaturalist and digitized herbarium specimen data in flowering plant families of the southeastern United States

**DOI:** 10.1371/journal.pone.0295298

**Published:** 2023-12-07

**Authors:** Elizabeth White, Pamela S. Soltis, Douglas E. Soltis, Robert Guralnick

**Affiliations:** 1 Department of Biology, University of Florida, Gainesville, Florida, United States of America; 2 Florida Museum of Natural History, Gainesville, Florida, United States of America; University of Tennessee at Chattanooga, UNITED STATES

## Abstract

iNaturalist has the potential to be an extremely rich source of organismal occurrence data. Launched in 2008, it now contains over 150 million uploaded observations as of May 2023. Based on the findings of a limited number of past studies assessing the taxonomic accuracy of participatory science-driven sources of occurrence data such as iNaturalist, there has been concern that some portion of these records might be misidentified in certain taxonomic groups. In this case study, we compare Research Grade iNaturalist observations with digitized herbarium specimens, both of which are currently available for combined download from large data aggregators and are therefore the primary sources of occurrence data for large-scale biodiversity/biogeography studies. Our comparisons were confined regionally to the southeastern United States (Florida, Georgia, North Carolina, South Carolina, Texas, Tennessee, Kentucky, and Virginia). Occurrence records from ten plant families (Gentianaceae, Ericaceae, Melanthiaceae, Ulmaceae, Fabaceae, Asteraceae, Fagaceae, Cyperaceae, Juglandaceae, Apocynaceae) were downloaded and scored on taxonomic accuracy. We found a comparable and relatively low rate of misidentification among both digitized herbarium specimens and Research Grade iNaturalist observations within the study area. This finding illustrates the utility and high quality of iNaturalist data for future research in the region, but also points to key differences between data types, giving each a respective advantage, depending on applications of the data.

## Introduction

The push to deinstitutionalize the acquisition of biodiversity occurrence data has greatly increased the breadth of data made accessible via participatory science initiatives [1–3]. Some of the most popular participatory science data platforms are apps and web-based programs in which users upload records of opportunistically observed living organisms, storing the time and location where the organism was observed (e.g., iNaturalist) [4]. These kinds of occurrence data have been studied and used in a wide variety of published research (e.g., [5–8]), but there is still considerable contention surrounding the quality of participatory science data and how it should be combined with expert-vetted natural history collection data in publishable research [9–12].

### iNaturalist as a data source

In iNaturalist, both observations and identifications are user-driven. Within this framework, a “Research Grade” observation is an observation that has more than two thirds of all identifiers agreeing on a species-level identification (with a minimum of two identifiers) [13]. Since 2012, these “Research Grade” observations have been routinely published in the Global Biodiversity Information Facility (GBIF) [14], which is a source of standardized occurrence data from multiple international sources, making these observations widely available for researchers to download and use in combination with other kinds of occurrence data.

Published studies using iNaturalist now cover a wealth of topics similar in scope to the applications of museum specimen data [3, 6, 7, 11, 15, 16]. The recent popularity and ready availability of participatory science data have also prompted researchers to develop protocols for the use of these data in various research applications, given a presumed higher level of “noise” in iNaturalist data that requires further filtering as compared to collections-based data [17].

Since the term “citizen science” (referred to here as “participatory science”, [18]) was coined in 1989 [19], there has been contention regarding metrics of data quality and accuracy of participatory science data. Recent studies have found moderate levels of misidentification in iNaturalist data, which has made researchers cautious of using participatory science data as a whole. McMullin [20] found that species that require microscopy or chemistry to make taxonomic distinctions most likely will not have the information required to make proper species delineations from participatory science observations, unless there are subtle morphological differences that allow species to be delimited without reference to the typical diagnostic characters. The latter approach still requires a priori knowledge of the taxonomy and biology of the organismal group and is less of a quantifiable process on a large scale.

### Museum collection data and herbarium digitization

With technological advances and rapid digitization, massive amounts of natural history collection data are available for aggregation and use in research. Herbaria in particular are crucial to the study and conservation of plants, as having a physical plant specimen preserved allows for fine-scale standardized morphological assessments, the possibility of using tissue for genetic study, and a variety of other advantages to research [21–23].

Studies assessing bias in the use of natural history collections have found issues not dissimilar to the concerns raised by some regarding participatory science data [24–27]. Possible biases include an overrepresentation of rare species [28], selective focus on certain groups or families depending on herbarium staff specialization, bias in collection locality (e.g., closer to cities or universities) [23, 29], and temporal biases associated with a general decline in botanical collections made in the last 100 years [30, 31].

iDigBio is a data aggregator that has been storing and serving digitized natural history collections and data since 2011 and today is a massive repository of occurrence data, with nearly 140 million digital records [32–34]. iDigBio, and other large data aggregators such as GBIF, are used often as a source of species occurrence data to perform large biodiversity analyses. Unlike GBIF, however, iDigBio does not incorporate participatory science data (such as iNaturalist observations).

Digitization of herbarium specimens has opened up a wealth of opportunities in the application and accessibility of herbarium data [21, 22]. Efforts to standardize the image format of digitized specimens [35] has improved their value for morphological studies. The use of digitized herbarium specimens in informing AI-driven models for plant identification, morphological, and phenological studies is of particular note [36–40], but having a better idea of the taxonomic error rates in varying groups of plants before using these models will be of utmost importance as a model informed by misidentified specimens could perpetuate bias and incorrect information [24]. Additionally, previous studies have assessed herbarium images by themselves on a large scale and found some complexity regarding taxonomic identification and accuracy, largely cases of outdated taxonomy, but in some cases misspellings or misidentifications [41].

### Present study

Advocates for the use of participatory science occurrence data argue that they provide a wealth of additional information that is often comprehensive and more current than natural history collection data [3, 6, 7, 42, 43], while skeptics raise issues regarding the potential inaccuracy of non-expert identification [10, 12, 13]. This study provides a direct comparison of the two kinds of data (participatory science vs. natural history collection) to permit a better understanding of both, as well as insights into if and where they differ in quality. Our study explores the limitations of using images as a data source to a novel extent and scale and provides insight into the processes of botanical taxonomic identification when only images are available.

In this study, digitized herbarium specimens were used as a proxy for herbarium specimens as a whole for the purposes of standardization of the image identification process. Numerous caveats related to this decision are addressed in more detail in the Discussion. Broadly, this decision emphasizes both the unique nature of the iNaturalist platform in that it operates solely on image identification, but also stresses the importance of incorporating ample images of distinguishing features into the standard herbarium digitization protocol moving forward, if the goal is ultimately to make these kinds of data comparable at large scales.

Recently published studies have investigated the quality of solely iNaturalist identification [13, 44]; here, we aim to provide a direct comparison of the proportion of misidentified observations by assessing iNaturalist and digitized herbarium data side by side. iNaturalist identification errors have been assessed in other studies, but these studies were either at smaller scales than our study or did not make any comparisons with digitized herbarium data [13]. We confine our scope to a case study among vascular plant families in the southeastern U.S. (confined by a bounding box with GPS localities (SW 24.629, -94.855, NE 38.017, -77.004), including Florida, Georgia, North Carolina, South Carolina, Tennessee, Kentucky, Alabama, Louisiana, Mississippi and parts of eastern Texas and southern Virginia) to keep all occurrences focused within a single flora. In addition, our approach helped to minimize taxonomic discrepancies caused by differing taxonomic opinions, as Weakley’s Flora of the Southeastern U.S. [45] is the most current reference, and is most comparable to the taxonomy that iNaturalist uses as the authority for vascular plants (Plants of the World Online) [17, 46, 47]. Previous assessments of bias and misidentification in participatory science data [48–50] have led us to hypothesize that the data quality of iNaturalist Research Grade observations will be substantially lower than for digitized herbarium specimens, with iNaturalist showing higher levels of taxonomic misidentification. We also hypothesize that there will be more observational bias towards common or visually charismatic plants and less evenness of observations in iNaturalist. Conversely, we expect to see a more evenly distributed representation of species in digitized herbarium collections.

## Methods

### “Identifiability by image” and focal family selection

The decision to investigate solely flowering plants was largely for the purpose of assessing taxa with differing levels of what we are describing here as “identifiability by image.” “Identifiability by image” refers to the accessibility of crucial structures for species-level identification to the average observer. In flowering plants, this most often means the prominence of reproductive structures when looking at the plant as a whole. This emphasis on reproductive characters ties into phenology, flowering time, and the taxonomic splitting of the group of plants at hand. For example, angiosperm families mainly composed of species with showy flowers are more likely to be collected/photographed and have enough information pictured or collected to be able to make a confident species identification than groups with inconspicuous flowers [51, 52]. In contrast, it is expected that families for which species delimitations often require fine-scale morphology, such as seed or trichome features, would be less likely to be photographed adequately for species identification [20]. Study of the biases regarding which kinds of plants are most photographed on iNaturalist and how this compares to what is most often represented in herbaria should be investigated further to fully understand the extent of these patterns. Here, we address the issue of “identifiability by image” solely as a means of selecting angiosperm families for further study. We view “identifiability by image” as a spectrum that may be observed for any given family or at any taxonomic level, and this concept has been explored previously in arthropod taxa [53]. A more comprehensive understanding of how groups vary in their ability to be identified by image is important in developing an understanding of the extent to which platforms such as iNaturalist can be used effectively in research, or to what extent digitized herbarium specimens can be used detached from their physically preserved specimens.

**Fig 1:**
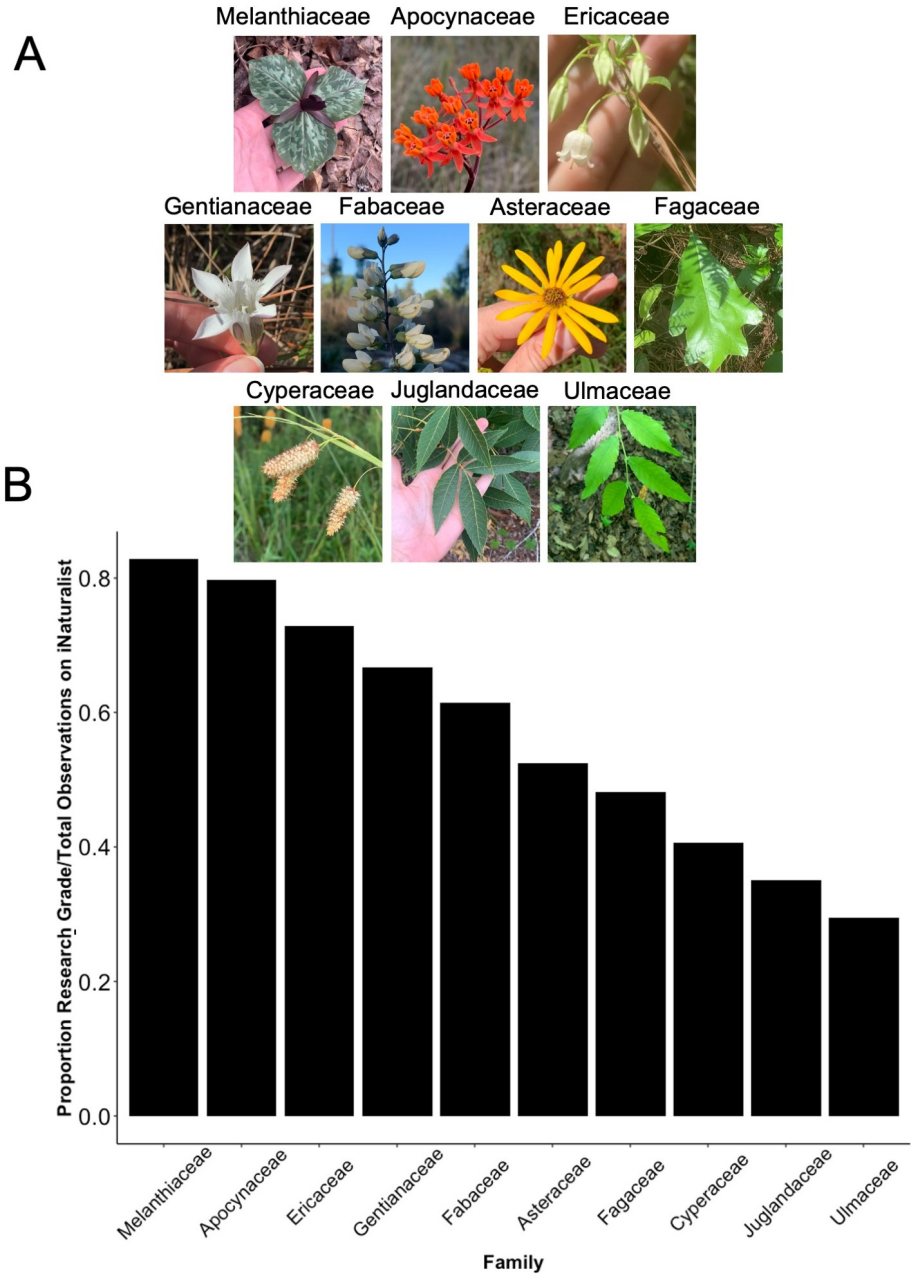
Identifiability by image and focal families. A. Representative images of each family included in this study. B. Selected angiosperm families based on proportion of Research-Grade/total observations on iNaturalist in the southeastern United States as a proxy for ease of “identifiability by image”/charisma. Images by Elizabeth White.

In our attempt to quantify this spectrum of “identifiability by image”, the iNaturalist identification process was used as a proxy, as iNaturalist specimens are only ever identified by available image and locality data. A dataset of all angiosperm iNaturalist observations available on December 31, 2021 from the southeastern U.S. was downloaded. The number of “Research Grade” and “Needs ID” iNaturalist records for each plant family was stored, and we first filtered out families that contained fewer than 500 Research Grade observations. Ten flowering plant families were then selected along a spectrum of 30% Research Grade to 85% Research Grade/Total observations to capture the spectrum of identification, using percentage Research Grade identification as a proxy for ease of “identifiability by image” (Fig 1).

### Occurrence download

**Fig 2:**
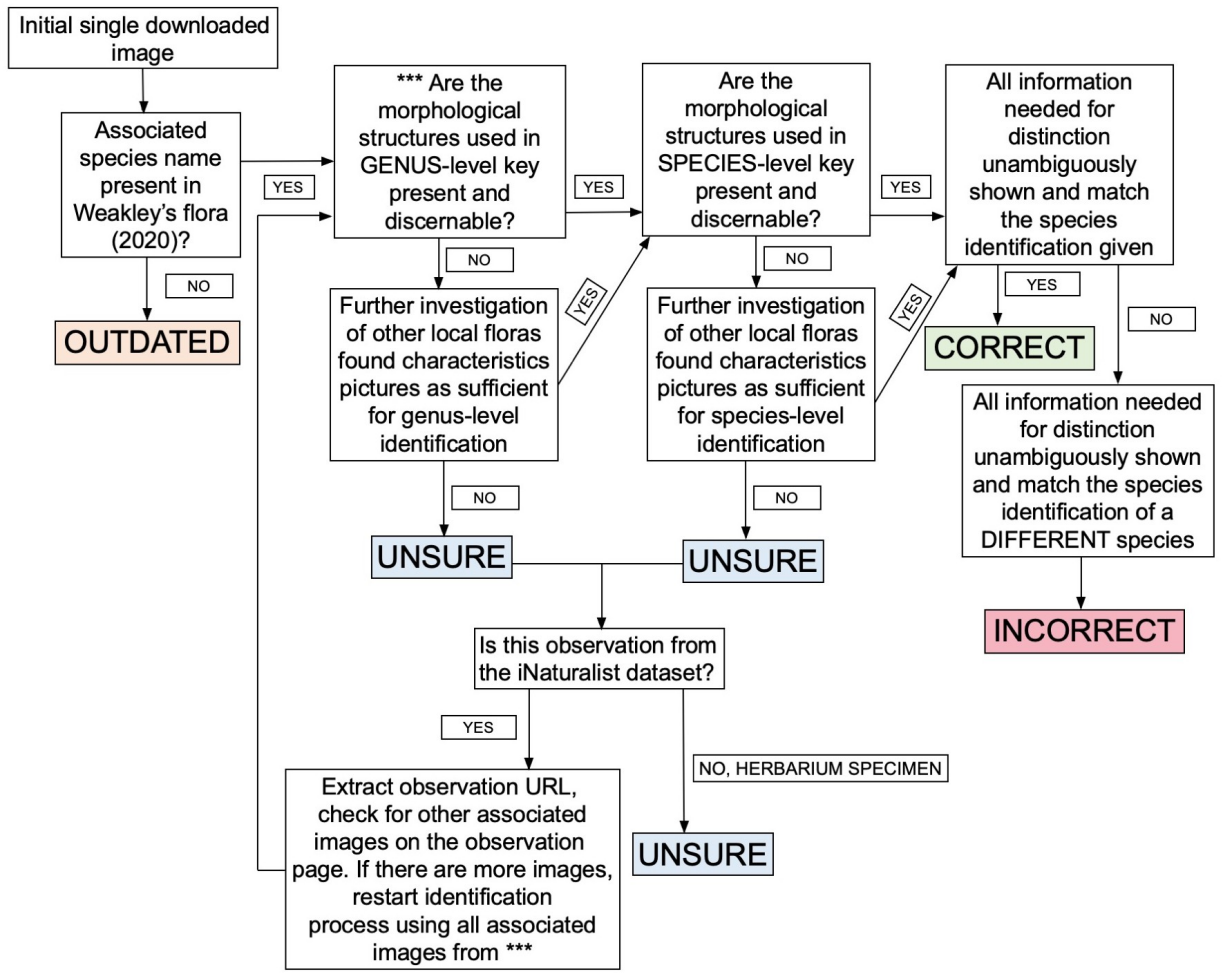
Decision tree/rubric for identification of digitized herbarium specimen images and iNaturalist photographs. Asterisks refer to other local floras used in “Unsure” specimens/observation delineation include Wunderlin [58], Wofford [59], and Barkworth [60].

Occurrence data for each of the ten selected families within the region covered by Weakley’s Flora [16] were downloaded using the iDigBio online portal (https://www.idigbio.org/portal/search) and from only Research-Grade observations via the iNaturalist “export observations” portal (https://www.inaturalist.org/observations/export). iNaturalist data were downloaded using the online export tool as opposed to isolating Research-Grade iNaturalist records from GBIF due to the more detailed data available (including identifier name, initial taxonomic identification, time until Research-Grade identification, whether the species is captive or cultivated, etc.). Names of species were only retained in the iDigBio dataset if those names were also present in the Plants of the World Online [46], the accepted taxonomic framework used by iNaturalist for vascular plants. This was done to reduce taxonomic discrepancies caused by outdated taxonomy, which is likely to be more prevalent in natural history collections and distracts from the question of data quality comparison in these two groups [54–57]. Images were first extracted from each occurrence record via associated image URLs found in iNaturalist and iDigBio occurrence downloads, and the associated taxonomic name was saved as the image file name. In the case of iNaturalist specimens identified as “Unsure” in the first pass of image identification, an additional round of identification occurred which included accessing each observation via URL to assess all associated images (Fig 2).

### Scoring images/identification calibration

Five hundred observations or records were randomly selected from each respective iNaturalist or iDigBio family data file using base functions within RStudio, resulting in 1000 specimens and Research-Grade iNaturalist observations for each of the 10 selected families. Associated images from all observations/specimens were downloaded and stored in folders by family and data type (“herbarium” or “inat”) to be scored using the image scoring software ImageAnt (https://gitlab.com/stuckyb/imageant). ImageAnt is an interface that allows each image in a folder to be assessed, displaying the image filename, the image itself, and a predetermined scoring system to be shown all at the same time, which allows images to be assigned scores quickly and stores score data to be easily analyzed in the future. In this case, the categories “Taxonomically Correct”, “Taxonomically Incorrect”, “Unsure”, “Outdated Taxonomy”, and “Flag” were used (Flag was rarely used and was applied to images in which the download did not work and an incorrect image was downloaded, to be revisited and corrected later), and each unique image set associated with an individual observation was independently identified according to the dichotomous keys found in Weakley’s Flora [16] and compared to the image filename that included the species determination given to the specimen or iNaturalist observation, to put each image in one of the aforementioned categories. It should be noted that a key distinction between the vast majority of digitized herbarium specimens is that they are associated with one downloadable image, whereas each iNaturalist observation is more often associated with multiple images. A representative image from each iNaturalist observation was initially used in identification scoring, and in cases in which there was not enough information pictured in the single downloaded iNaturalist image, the observation was marked as “Unsure”, and then observation URLs were extracted for each observation. Identifications were manually scored based on the set of all images associated with the observation (Fig 2).

Taxonomic identification was calibrated using a group of expert botanists and taxonomists who were assigned the same group of images at the beginning of the project, to come to a consensus of what constitutes an “Unsure” image, the most ambiguous and subjective scoring category. It was decided that images with no reproductive structures pictured and that also had no mention of leaf characteristics in the family’s available dichotomous keys would be marked in the “Unsure” category. After this identification framework was created and identifications were calibrated, downloaded images were scored using Weakley’s Flora and our developed identification framework (Fig 2).

### Statistical methods

After all images were scored, “Correct” and “Unsure” identifications were summed for each data type, and a two-proportions Z-test was run using the stats package in R for each to determine the effect of data type (iNaturalist or Herbarium) on taxonomic identification. Further nuance on the inclusion or omission of the “Outdated” category is discussed further below.

To test the extent of variation in error rates across taxonomic groups, we fit two separate binomial general linear models (one GLM for iNaturalist and one for digitized herbarium records) with correct versus incorrect as response, and family as a covariate. We compared the model with family as a covariate to an intercept only model, and used an ANOVA analysis to test the importance of family as a predictor. Visualization of statistical models was constructed using the R packages sjPlot and ggPlot [61, 62].

To test coefficient of variance as a metric of evenness in observation per species in the iNaturalist and herbarium datasets, we used the dplyr package in R to create a vector of “counts” for how many times each species was observed in the entire data download for each data type [63]. Then, we used the stats package in baseR to calculate mean and standard deviation of each “counts” vector. The coefficient of variance was calculated for each dataset manually in R by dividing the standard deviation by the mean and multiplying by 100.

## Results

### Data type comparison

**Fig 3:**
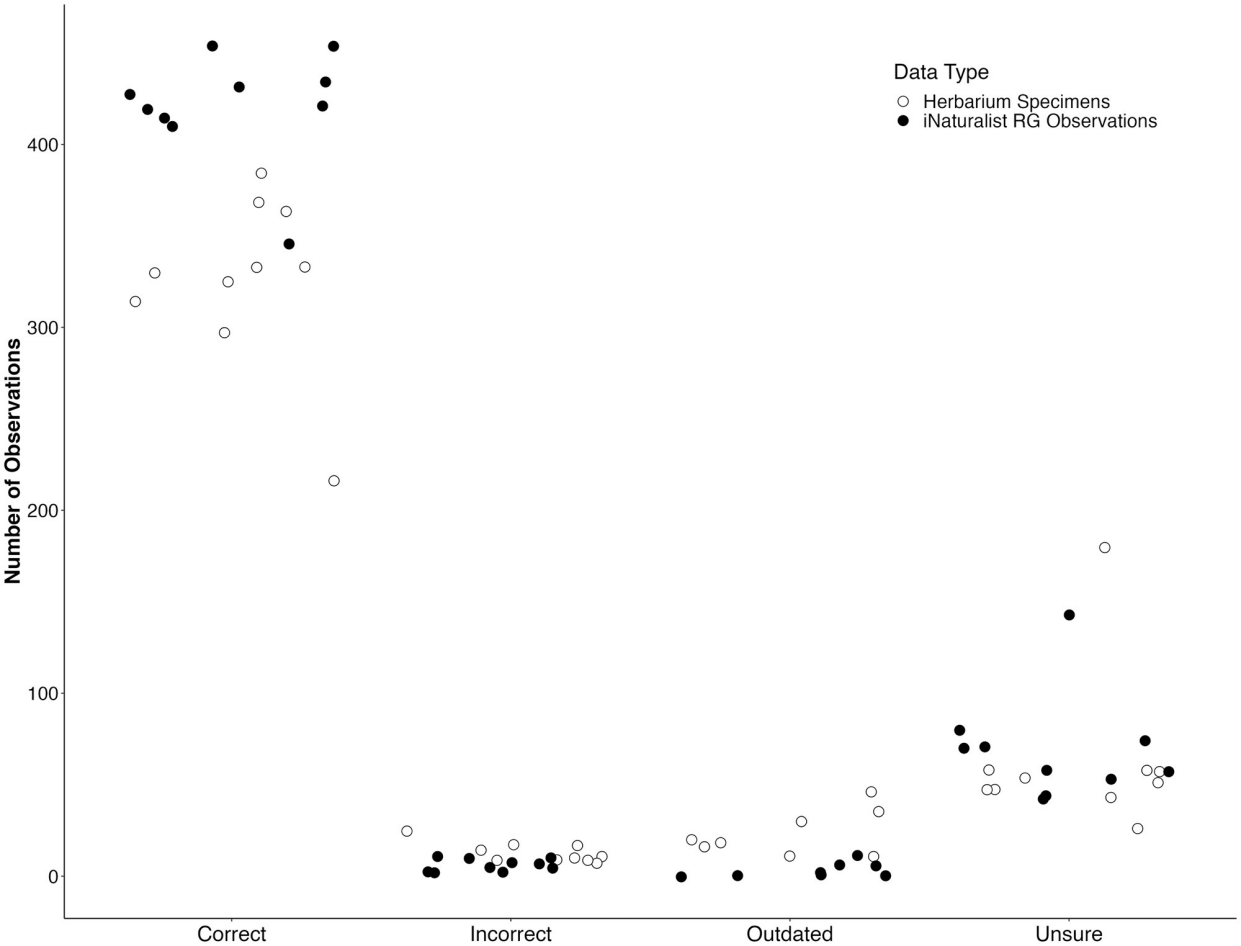
Total number of Correct, Incorrect, Outdated, and Unsure taxonomic identifications for digitized herbarium specimens (white dots) and iNaturalist (black dots). Each dot represents a sampled plant family.

Two two-proportions Z-tests were run to determine the effect of “data type” (iNaturalist or digitized herbarium specimen) on the frequency of each taxonomic identification. One Z-test was run to determine the effect of data type on the number of “Correct” identifications, and then the same process was followed for the “Unsure” category (Table 1). Data type was significant in predicting whether each observation/specimen was identified correctly (p <0.001), with iNaturalist having higher accuracy overall (iNaturalist proportion Correct = 0.84267, Herbarium proportion Correct = 0.76345). Data type was not significant in how many observations were placed in the “Unsure” taxonomic identification category (p = 0.3659), and digitized herbarium images were placed in this category at a higher frequency than scored iNaturalist images (iNaturalist proportion Unsure = 0.13851, Herbarium proportion Unsure = 0.14530). There were 113 cases in which iNaturalist observations were initially scored as “Unsure” and were then changed to a different category after assessment of all images associated with the observation URL (see S1 Dataset). Data type was also significant in predicting how many observations were placed in the “Outdated” and “Incorrect” categories, although in both cases the number of observations in these categories were comparatively low (Fig 3, Table 1).

**Table 1:** Two-proportions Z- test results showing effects of data type on how many observations were placed in the “Correct” or “Unsure” categories (Prop 1: iNaturalist, Prop 2: Herbarium).

	Two proportion Z- test results: Difference of each score category between iNaturalist and digitized herbarium images		
	p	Sample estimates	95% confidence interval
**Correct**	2.2e-16 ***	Prop 1: 0.84267	0.06274777
		Prop 2: 0.76345	0.09569363
**Unsure**	0.3	Prop 1: 0.13851	-0.02126412
		Prop 2: 0.14530	0.007691451
**Outdated**	2.2e-16 ***	Prop 1: 0.00578	-0.04017332
		Prop 2: 0.03921	-0.02669221
**Incorrect**	2.2e-9 ***	Prop 1: 0.01334	-0.02646847
		Prop 2: 0.03302	-0.01288861

#### Between-family analysis (General Linear Models)

**Fig 4:**
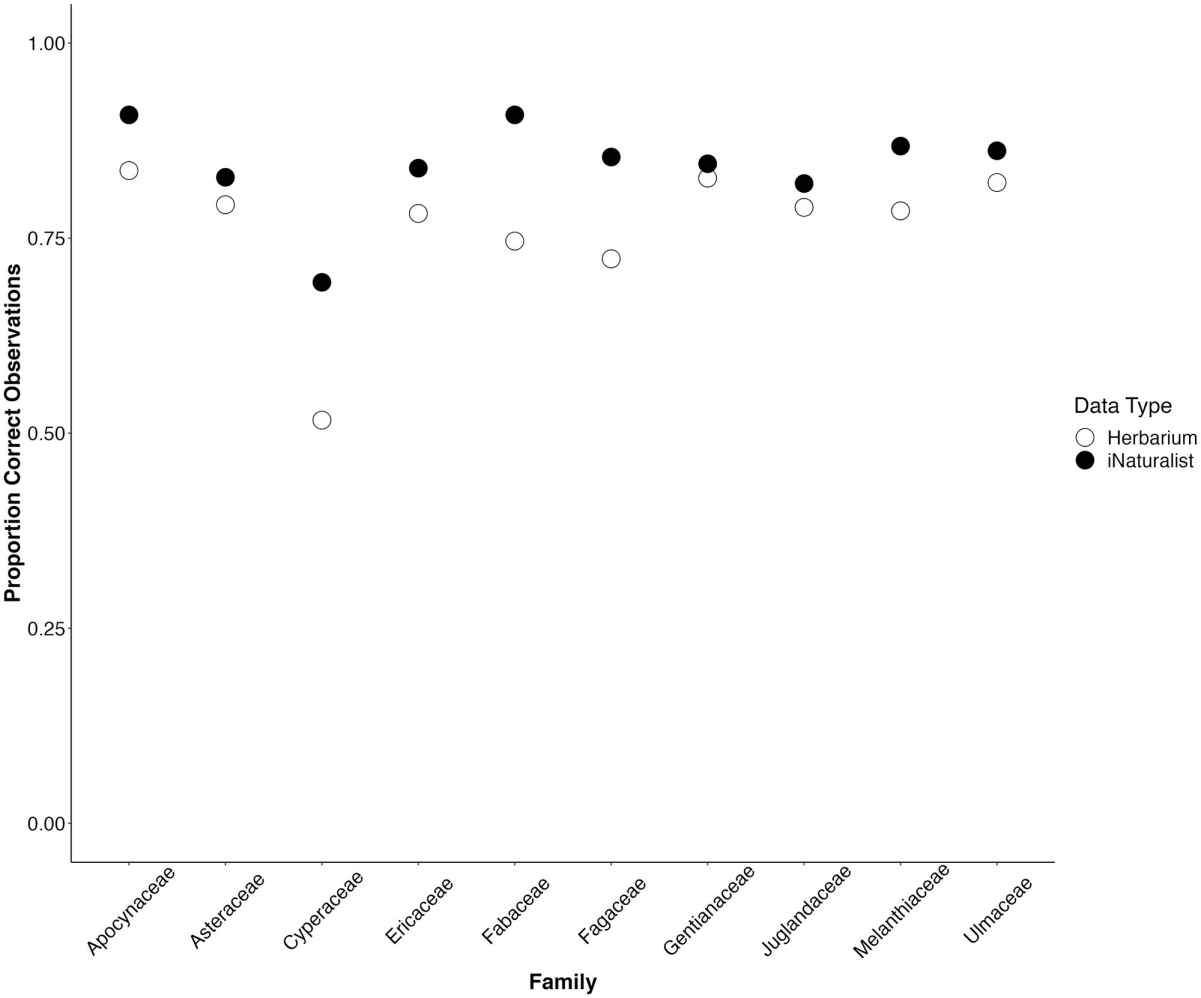
Proportion Correct identifications across families for digitized herbarium specimens and iNaturalist. Dots represent the correct number of correctly identified observations divided by the total number of observations downloaded per family (500).

Results of the GLM analyses showed that plant family had a significant effect on the accuracy of taxonomic identification. In both cases, the model with family is significantly better than an intercept-only model (p < 2.2 e -16 in all cases, Table 2). This pattern is strongly driven by the high proportion of the family “Cyperaceae” being scored as “Unsure”, creating a low proportion of “Correct” identifications in Cyperaceae in both data types (Fig 4). We also plot “Correct”, “Incorrect”, “Outdated”, and “Unsure” by family in Fig 3 to visually summarize those distributions.

**Table 2:** ANOVA family-level results. Testing General Linear Models against null models to determine family effect on taxonomic identification.

	Residual df	Dev df	Pr(*<0.05)
**Model 1:** **iNaturalist taxonomic identification ~ Family**	4952	4107.9	p < 0.001 ***
**Model 2:** **Digitized Herbarium taxonomic identification ~ Family**	4002	3645.7	p < 0.001 ***

### Analysis of evenness of species representation/observation number (coefficient of variance)

**Fig 5:**
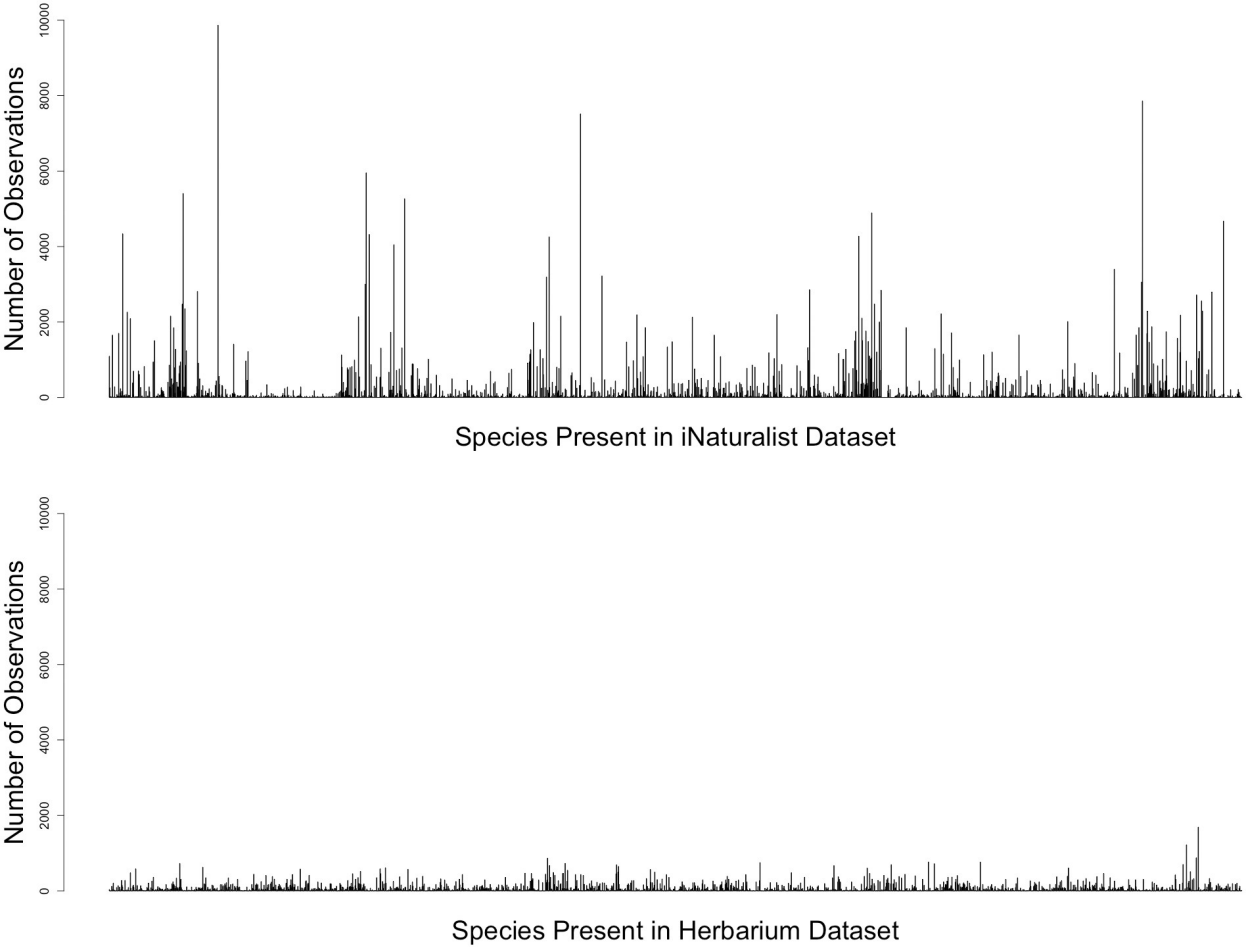
Histograms showing distribution of number of observations for each species across all families in this downloaded dataset. iNaturalist (n = 2676 species) (above) and digitized herbarium specimens (n = 2468 species) (below).

Results of an analysis of coefficients of variance on the total dataset of all plant observations and specimens from the 10 selected families and within the study area (409,636 iNaturalist records, 194,662 digitized herbarium records), showed that iNaturalist had higher variance (332.32%) when accounting for the mean number of observations per species than digitized herbarium specimens (181.22%). The number of observations per species in the digitized herbaria dataset is more centralized around the mean (64.82), whereas iNaturalist observations are less evenly centered around the mean number of observations per species (152.77). This result further suggests more evenness of observation in the digitized herbarium dataset and generally large peaks in observations of a few species in the iNaturalist dataset (Fig 5).

## Discussion

We show that there are some modest differences in the proportion of correctly identified specimens between iNaturalist Research-Grade observations and digitized images of herbarium specimens across flowering plants in the southeastern U.S. for the families investigated, which are largely representative of the diversity in angiosperm families. In general, iNaturalist Research-Grade specimens have slightly greater odds of being identified correctly compared to digitized herbarium specimens. However, different identification processes for the two types of data may affect identification accuracy and bias.

### Caveat: Use of digitized herbarium specimens

Both herbarium specimen images and iNaturalist images are ultimately tied back to collecting events. At the time of collection, identifications are often made based on observation of the physical organism, and therefore identification may include information that is not captured on images available to digital data users. This should be kept in mind in the context of interpretation of this study’s results. We still see value in the comparisons here, both to assess quality based on available evidence from images, and to better understand how these types of data can be comprehensively and effectively combined by the common factors that unite them, their identification to comparable taxa and their availability as digital objects.

The decision to analyze digitized herbarium specimens as opposed to physical herbarium specimens was for the reasons of ease of data accessibility and framing of the questions at hand. Given that we were looking at the utility and behavior of images as a taxonomic tool and the limits of imaging plants as a medium, the inclusion of physical museum specimens would not be testing the same phenomena as the iNaturalist observations. Digitized specimens are also standardized in their format, which brings advantages and disadvantages: the results of this study point to the utility of the freedom that iNaturalist observers have to upload multiple images of morphological structures all associated with the same observation, whereas a digitized herbarium specimen is nearly always only one image. This would not be the case with physical herbarium specimens that one could examine in otherwise fine detail (although certain morphological characters may be lost or degraded through pressing, drying, and mounting).

The “Unsure” category includes two different phenomena: 1) images that provided little to no information for identification past the family or genus level due to degradation of the specimen or poor image quality, and 2) images of species that require fine-scale morphology, chemistry, or chromosome number to distinguish among species (Fig 2). Here, we treated both types similarly, but the distinction is worth noting when thinking about the implications of this concept of “identifiability by image”, as there is a difference between issues that arise by identifying organisms when only images are available as compared to the issues that relate more to the standardization of images being used for identification, as some taxonomic groups require more detailed images than others and some distinguishing characters by definition (as in the case of chemistry or chromosome number) cannot be imaged.

The results for digitized herbarium specimens of Cyperaceae imply a more prevalent issue of the limits of the standardized herbarium digitization protocols used today. The results of this study call for incorporation of the extended specimen protocol into standardized digitization efforts (including close-up or microscope images where appropriate) when of particular relevance to identification. iNaturalist provides more flexibility for those users with prior knowledge of a particular taxonomic group by allowing them to provide more detailed images (although this does not happen frequently enough, as quantified by McMullin [20]). Still, iNaturalist observations used in this study did include microscope or magnified images of distinguishing characters, hence the higher proportion of Correct scorings for iNaturalist than digitized herbarium specimens for taxonomically complex families such as Cyperaceae. Although multiple images of a herbarium specimen can often be accommodated by the database structure, standard digitization protocols nearly always capture only a single image of the entire specimen, and although some images are of sufficient quality for zoomable close-ups, multiple images, including close-ups, are rarely available [64–66]. This practice, and the ensuing results, also stress the importance of the availability, accessibility, and preservation of the physical specimens in natural history collections, as some groups of organisms do not lend themselves to the identified-by-image-only system.

### Identification accuracy

A main difference in these two types of data is the overall standardization of both the intake and identification processes in iNaturalist relative to herbarium specimens. With iNaturalist, photographs are submitted to the platform by participatory scientists, and all photographs are then open for other iNaturalist users to help refine initial identifications or make other annotations that increase the utility of the photograph for research value. In contrast, images of herbarium specimens come from multiple sources via multiple platforms that use diverse pipelines. For example, the download of data from iDigBio (or any other data aggregator) could be biased due to lags in herbarium digitization efforts and upload time to the iDigBio database from individual herbaria.

In addition, taxonomic discrepancies and outdated taxonomy are major setbacks to the standardization of herbarium data from multiple institutions and even potentially within a single collection. Objects (digital or physical) that have not been recently reassessed are more likely to be represented in herbarium data, and this issue lends itself to problems of synonymy. By contrast, a single, consistently updated taxonomic backbone underlies iNaturalist. This standardization means that issues with synonymy are much less common in iNaturalist.

Caution should be exercised when using iNaturalist Research-Grade data without ample distinguishing characters pictured, specifically for species identifications that require fine-scale morphology (e.g., Cyperaceae, Poaceae, many bryophytes). It should be noted, however, that iNaturalist is a platform in which anyone can identify images, and identifiers are experts in their respective fields, whether classically trained taxonomists or extremely knowledgeable and experienced participatory scientists who have an intimate understanding of certain groups of organisms [43, 67]. Even in cases when only images are available, it is possible in the future that computer vision or comparable image recognition software would be able to make accurate predictions based on other characters in images that may not be able to be picked up by someone using a traditional flora, if enough high-quality identifications already exist for the group and are informing the given identification software. This requires further investigation in the future, as the Research-Grade identification process on iNaturalist heavily relies on user identifications as it stands today [43].

### Differences in process and bias

Computer vision has significantly altered the process of observation and identification in iNaturalist [68]). For iNaturalist users who have no prior knowledge of the taxonomic group they are posting, the computer vision-generated identification is likely what the initial, (non-Research-Grade) identification will be associated with. Many concerns with identification accuracy come from this part of the identification process, implying that identifiers on iNaturalist are more likely to accept an erroneous AI-generated identification than a trained expert doing the identifying in a natural history museum, particularly in more difficult-to-identify groups or groups of species that are variable in the morphology. However, recent work shows that computer vision may have less impact on downstream identification processes—past the initial observation—than has been assumed, and generally that the computer vision framework has been shown to have significant utility in reducing the time it takes to get to Research Grade when used for the first identification associated with an iNaturalist observation [43].

Our results also indicate an overrepresentation of species based on relative abundance and charisma. Members of Cyperaceae are better represented in the digitized herbarium dataset than in iNaturalist in the southeastern U.S., but in all other families, iNaturalist has more observations than what is available as imaged specimens in digitized herbaria. A search of the family “Cyperaceae” on the iDigBio portal for the study area in October 2023 showed 12,848 records present that did not have media attached, meaning that they exist in the collection but the specimen has not been imaged. Although iNaturalist has a higher abundance of observations overall than what is available in the digitized herbarium specimen dataset for the southeastern U.S. for the subset of families selected (409,636 observations in the iNaturalist dataset vs. 194,662 in the herbarium dataset), the iNaturalist data are less evenly distributed across species and generally have large peaks of observations in common species. Species that are common in disturbed areas such as lawns, ditches, or landscaped areas tend to have a much higher proportion of observations than other plant species (as is the case for *Bidens alba* in this dataset, which can be seen as the large peak in the upper panel of Fig 5). Similarly, in taxonomic groups (whether that be families or genera) that are generally difficult to identify by image, such as Cyperaceae, the vast majority of iNaturalist observations are centered around species that are particularly showy and that have large bracts or colorful reproductive structures (as is the case in the southeastern U.S. with *Rhynchospora colorata*), whereas in the digitized herbarium specimen dataset, more diverse and cryptic genera are more commonly represented (*Carex*).

### Implications for future research and a framework for future use

The use of iNaturalist data in biodiversity research is a promising field with the potential to become a massive source of occurrence data. Nonetheless, a better understanding of the identification process, as well as the biology of the organisms being examined with this kind of big data, should be taken into account more directly when considering data sources in large-scale biodiversity analyses. Caution should be exercised in the use of species occurrences based on images alone in groups that require fine-scale morphology, chemistry, chromosome number, etc. to make species-level identifications; this situation requires some a priori understanding of the biology and taxonomy of the group at hand. Ultimately, further studies of error and bias in species identification, building on this study and encompassing more families in a wider geographic scope, will be useful in capturing possible patterns of misidentification and bias of iNaturalist data more broadly. However, the results of this study reinforce the quality and accuracy of participatory science data to be used at large scales alongside occurrence data acquired from natural history collections.

## Supporting information

S1 Dataset Changed “Unsure” observations. List of iNaturalist observation URLS which were changed from “Unsure” to another identification category after assessing all associated images.
(XLSX)
